# Zoonotic potential of bovine *Sarcocystis* species: a challenge for public health and meat inspection

**DOI:** 10.1590/S1678-9946202668049

**Published:** 2026-07-24

**Authors:** Cayuan Tadeu Brandão Pinto, Luciana Regina Meireles

**Affiliations:** 1 Universidade de São Paulo, Faculdade de Medicina, Instituto de Medicina Tropical de São Paulo, Laboratório de Protozoologia (LIM-49), São Paulo, São Paulo, Brazil

**Keywords:** *Sarcocystis* spp, Zoonosis, Food safety, Sarcocystis sigmoideus, One Health

## Abstract

Foodborne parasitic diseases remain a neglected public health issue worldwide. Sarcocystosis, caused by the ingestion of tissue cysts of *Sarcocystis* species, represents a growing challenge for food safety and public health. Cattle serve as intermediate hosts for several *Sarcocystis* species, including those with confirmed zoonotic potential such as *S. hominis, S. heydorni*, and the recently described *S. sigmoideus* (2024/2025). In humans, intestinal sarcocystosis often presents with gastrointestinal distress, whereas extraintestinal (muscular) infections can lead to severe clinical manifestations, including acute eosinophilic myositis and, rarely, neurosarcocystosis. Despite these risks and the high epidemiological prevalence in beef herds worldwide, current sanitary inspection methods in abattoirs are primarily based on macroscopic visual examination. Said approach frequently fails to detect microscopic cysts, allowing the latent transmission of these pathogens. This review addresses the biology, clinical manifestations across different hosts (humans, bovines, and equines), and epidemiology of bovine sarcocystosis. We discuss the limitations of conventional morphological diagnosis and the critical need for molecular tools to accurately differentiate zoonotic from non-zoonotic species. Finally, we highlight the need for adopting a One Health approach, integrating sensitive diagnostic methods into the meat production chain to mitigate the risk of human infection and ensure global food safety.

## INTRODUCTION

Foodborne parasitic diseases pose a growing challenge to global public health, a scenario exacerbated by the increasing international trade concerning products of animal origin^1-3^. Brazil occupies a central position in this context as one of the world's largest red meat suppliers^4^. But this commercial leadership imposes a critical responsibility regarding sanitary surveillance, as beef can serve as a vehicle for several neglected zoonotic pathogens.

Although historically considered primarily a matter of animal health and economic loss within the production chain, bovine sarcocystosis has direct implications for food safety^5,6^. *Sarcocystis* spp. presents a high prevalence in cattle herds and is frequently underestimated. The public health risk lies in the difficulty of distinguishing between low-risk species to humans, such as *S. cruzi* (pathogenic only to cattle), and proven zoonotic species, such as *S. hominis* and *S. heydorni*, for which humans serve as definitive hosts^5,7^. Additionally, recent description of new species, such as *S. sigmoideus*, and the recognition of their zoonotic potential underscore the need for a One Health approach to monitor this parasite^8^.

Thus, ensuring the safety of Brazilian beef transcends the product's economic value. This article reviews the biological and epidemiological aspects of *Sarcocystis* spp., focusing on zoonotic and emerging species and discussing the limitations of current diagnostic methods and the implications of these gaps for public health and tropical medicine.

### General aspects, morphology, and life cycle

#### Historical background

Sarcocystosis was first described in the late 19th century when characteristic cystic structures were observed in the muscle tissues of infected animals. A researcher in Switzerland produced initial descriptions of the structure and organelles of *Sarcocystis* spp. in 1843, noting long, thin, white cysts in the musculature of *Peromyscus* rodents^9^. Importantly, these early 19th-century reports primarily described the morphological presence of the organism (initially termed "Miescher's tubules") in muscle tissue, rather than the clinical disease. For years, *Sarcocystis* spp. was believed to be a fungal organism. This misconception arose because an attempt to isolate it in cell culture resulted in contamination by filamentous fungi. It was not until 1967, with the aid of electron microscopy, that the tissue cysts found in the musculature of parasitized hosts were confirmed to be morphologically similar to those of the phylum Apicomplexa. Presence of the apical complex definitively ruled out any association with fungal taxonomy. Consequently, subsequent studies identified *Sarcocystis* spp. as obligate intracellular protozoa belonging to the phylum Apicomplexa, possessing a heteroxenous life cycle that involves intermediate (herbivores) and definitive (carnivores) hosts^10,11^.

In Brazil, sarcocystosis gained prominent relevance due to its high prevalence in cattle herds and its significant impact on meat production. Recent research indicates that the infection is vastly underdiagnosed, especially in asymptomatic animals, which hinders the implementation of effective control measures^12^. Moreover, the condemnation of contaminated carcasses has become a growing challenge for the meat industry, reinforcing the critical need for enhanced sanitary surveillance and public health monitoring^11,12^. Supplementary Table S1 summarizes the chronological timeline of *Sarcocystis* spp. identification and characterization.

#### Morphology and ultrastructure of main species

With over 200 species described in the literature, accurately identifying *Sarcocystis* spp. remains a constant challenge in the scientific community. Precise identification is vital for understanding their pathogenesis, epidemiological aspects, economic impact, and their role in Public Health and veterinary medicine^13^. Although macroscopic inspection notoriously fails to detect the microscopic tissue cysts of this parasite, and molecular methods are currently indispensable for DNA detection, the ultrastructural analysis of the tissue cyst walls provides crucial insights into a unique morphology. This enables the differentiation of species that would otherwise be indistinguishable under light microscopy^14^.

**Figure 1:**
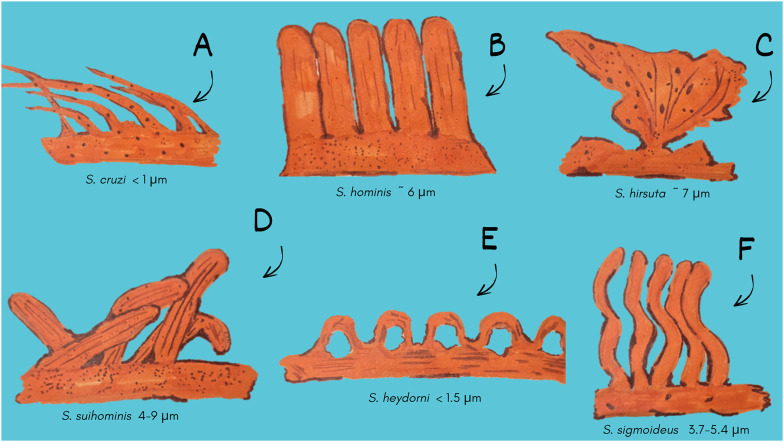
Schematic representation of the tissue cyst wall ultrastructure of six *Sarcocystis* spp. For orientation purposes across all panels, the villar protrusions project from the primary cyst wall toward the host tissue, whereas the basal (granular) layer is oriented toward the cyst interior. The illustrations highlight the morphological diversity of the villar protrusions, a crucial taxonomic characteristic for species differentiation: (A) *Sarcocystis cruzi*: Thin wall (< 1 μm) with long, narrow, ribbon-like villar protrusions that fold over the cyst surface (Type 7a); (B) *Sarcocystis hominis*: Thick wall (up to 6 μm) with long (approximately 7 μm), robust, cylindrical or finger-like villar protrusions (Type 10b); (C) *Sarcocystis hirsuta*: Thick wall (up to 7 μm) with villar protrusions that expand laterally and taper distally, frequently folded (Type 28); (D) *Sarcocystis suihominis*: Thick wall (4–9 μm) with very long villar protrusions (up to 13 μm), frequently slanted and disorganized in appearance (Type 31b); (E) *Sarcocystis heydorni*: Thin wall (< 1.5 μm) with short (approximately 0.5 μm), conical, and truncated villar protrusions; (F) *Sarcocystis sigmoideus*: Thick wall (3.7–5.4 μm) with densely packed, flattened, and undulating villar protrusions, exhibiting a characteristic sinuous "S" shape. Figure based on morphological data from Dubey *et al*.^14^, Dubey *et al*.^7^, and Rubiola *et al.* ^12^.

Thus, these ultrastructural characteristics are crucial, particularly concerning species with zoonotic potential and/or distinct species that impact the beef production chain. Among the *Sarcocystis* spp. that parasitize cattle and/or have zoonotic relevance, *S. cruzi* stands out as a species of great importance in veterinary medicine. It is highly prevalent in cattle and utilizes dogs as definitive hosts, forming microscopic tissue cysts (<1000 μm). Its cyst wall, less than 1 μm thick, is characterized by elongated, narrow, ribbon-like villar protrusions that frequently fold over the surface, granting it a type 7a appearance (Figure 1A). Conversely, *S. hominis*, recognized for its zoonotic potential as it uses humans as definitive hosts, possesses tissue cysts with thicker walls (up to 6 μm) and cylindrical or finger-like villar protrusions, approximately 7 μm in length, classified as type 10b. Notably, these protrusions contain numerous microtubules extending to the apex (Figure 1B).

The species *S. hirsuta*, which uses cats as definitive hosts, also exhibits thick walls (up to 7 μm) and villar protrusions that expand laterally in the middle and taper distally. These protrusions are about 8 μm long and are often folded at a 45–90 degree angle. Its ultrastructure is classified as type 28 (Figure 1C). *S. suihominis* also deserves highlighting. Although it is associated with pork, *S. suihominis* possesses zoonotic potential, utilizes humans as definitive hosts, and is frequently compared to bovine species. The sarcocyst of this species has a 4–9 μm-thick wall with elongated protrusions (up to 13 μm), classified as type 31b (Figure 1D).

*S. heydorni*, the zoonotic relevance of which was reaffirmed in 2015 after its initial description in 1973 via self-infection, has often been morphologically confused with *S. hominis* due to the similarity of their microscopic cysts in beef. But unlike *S. hominis, S. heydorni* forms tissue cysts with a thin wall (less than 1 μm thick) under light microscopy and a total thickness of 1 to 1.5 μm under electron microscopy. Regarding its ultrastructure, its cyst wall exhibits short, conical villar protrusions measuring up to 0.5 μm in length and width^7^. While the reviewed literature currently lacks a standardized schematic diagram of the ultrastructural wall for *S. heydorni*, its features are similar to type 29a (Figure 1E).

Finally, *S. sigmoideus*, a recently described species (2024) isolated from bovine muscle in Italy and confirmed in 2025 as a zoonotic species with humans as definitive hosts, presents cysts with thick walls (3.7–5.4 μm). These walls feature densely packed, flattened, undulating, and narrow protrusions that exhibit a characteristic S-shape when observed laterally. The base of these protrusions is narrower (100–150 nm), containing fine microtubules that extend from the base to the apex without, however, protruding into the thin layer of ground substance (Figure 1F)^8^. Despite no standardized schematic diagram of the ultrastructural wall available in the reviewed literature for *S. sigmoideus*, descriptions based on transmission electron microscopy have already revealed a singular morphology, making this species a promising target for future taxonomic studies and molecular diagnostics.

Figure 1 and Table 1, respectively, schematically illustrates and summarizes the morphological diversity of the tissue cyst walls, which serves as a fundamental taxonomic tool. These representations were created based on the ultrastructural illustrations consolidated by Dubey *et al*.^14^ and the most recent publications describing the walls of *S. heydorni* ^7^ and the newly described *S. sigmoideus* ^8^.

**Table 1:** Morphological and biological characteristics of key *Sarcocystis* species affecting cattle, humans, and equines

*Sarcocystis* species	Intermediate host(s)	Definitive host(s)	Cyst wall thickness (Light microscopy)	Cyst wall morphology (TEM) types	Primary tissue tropism	Zoonotic potential	Article
*S. cruzi*	Cattle	Canids	Thin (< 1 μm)	Type 7a (Ribbon-like villar protrusions)	Cardiac / Skeletal Muscle	No	Fayer^10^ Rosenthal^11^ Dubey *et al*.^14^
*S. hominis*	Cattle	Humans / Primates	Thick (up to 6 μm)	Type 10b (Cylindrical villar protrusions)	Skeletal Muscle/Cardiac Rare	Yes (Intestinal)	Rosenthal^11^ Dubey *et al*.^14^ Pena *et al*.^34^
*S. hirsuta*	Cattle	Felids	Thick (up to 7 μm)	Type 28 (Laterally expanded protrusions)	Skeletal Muscle	No	Dubey *et al*.^14^ Moré *et al.* ^30^
*S. heydorni*	Cattle	Humans	Thin (< 1 μm)	Type 29a-like (Short, conical protrusions)	Skeletal Muscle	Yes (Intestinal)	Dubey *et al*.^5^ Dubey *et al*.^7^
*S. sigmoideus*	Cattle	Humans	Thick (3.7 – 5.4 μm)	Undulating / S-shape protrusions	Skeletal Muscle (Assoc. with BEM)	Yes (Intestinal)	Moniot *et al*.^8^ Rubiola *et al.* ^12^
*S. neurona*	Accidental: Equines, Marine mammals	Opossums	N/A (Does not encyst in accidental hosts)	N/A	Central Nervous System	No	Reed et al.^25^
*S. nesbitti*	Accidental: Humans, Monkeys	Snakes	Unconfirmed in humans	Unconfirmed	Skeletal Muscle/Central Nervous System	Yes (Muscular / Neural)	Lau *et al*.^15^ Kwok *et al*.^18^ Skarpengland *et al*.^19^ Italiano *et al.* ^44^

As new species of *Sarcocystis* spp., such as *S. sigmoideus*, are discovered and described in the literature^8^, the need for continuous updates in review studies becomes paramount. This requirement extends beyond merely focusing on diagnostic methods; it must also encompass the visual representation of the ultrastructural features that differentiate these parasite species. The lack of standardized ultrastructural diagrams and the pressing need for schematic illustrations for *S. heydorni* and *S. sigmoideus*, respectively, highlight a fertile ground for future research and the enhancement of didactic resources in parasitology.

#### Transmission and life cycle

Human sarcocystosis transmission occurs via two distinct epidemiological routes: (i) ingestion of meat and its by-products containing tissue cysts with viable bradyzoites; and (ii) ingestion of oocysts or sporocysts containing viable sporozoites. The latter are excreted in the feces of definitive hosts and easily disseminate in the environment, contaminating water sources and raw foods such as fruits and vegetables^11^. When human infection results from the ingestion of tissue cysts, the individual develops the intestinal form of the disease, acting as the definitive host for the parasite. Conversely, the accidental ingestion of oocysts or sporocysts leads to the muscular form of the disease, in which humans act as intermediate hosts. Notably, species with proven zoonotic potential responsible for human intestinal sarcocystosis include *S. hominis, S. heydorni*, and the newly described *S. sigmoideus*, confirmed as zoonotic via the consumption of beef, and *S. suihominis*, which is transmitted via pork^8-12^. Additionally, humans can act as accidental intermediate hosts for *S. nesbitti*, the primary etiologic agent of human muscular sarcocystosis. Initially described in the musculature of non-human primates (*Macaca mulatta*), molecular methods have corroborated the epidemiological importance of this species in human outbreaks of the disease^15^.

**Figure 2:**
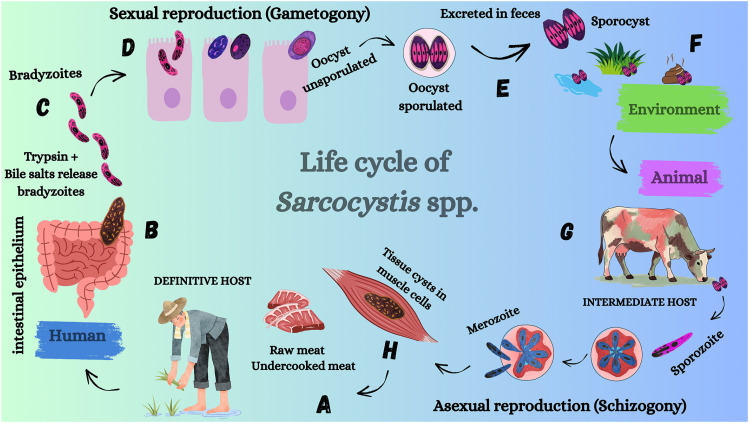
Life cycle of *Sarcocystis* spp.: (A) The cycle begins with the ingestion of raw or undercooked meat containing tissue cysts by a definitive host (DH); (B) In the intestinal lumen of the DH, the action of trypsin and bile salts promotes cyst rupture; (C) releasing infective bradyzoites. These invade the enterocytes of the intestinal epithelium, where they differentiate and initiate; (D) gametogony (sexual reproduction), forming macro- and microgametes that fuse to originate the zygote (2n); (E) The zygote develops into an unsporulated oocyst, which sporulates within the tissue itself, forming two sporocysts, each containing four sporozoites; (F) Oocysts or free sporocysts are excreted in the feces, contaminating the environment; (G) An intermediate host (IH), such as cattle, becomes infected by ingesting contaminated water or pasture. In the IH, the sporozoites initiate schizogony (asexual reproduction), undergoing two generations of development (schizonts) in endothelial cells, releasing merozoites. The merozoites invade muscle and/or nerve cells, forming tissue cysts. Initially, the immature cyst contains metrocytes (non-infective), which multiply and give rise to bradyzoites, (H) forming the mature and infective cyst, which can be ingested by a new DH, completing the cycle. Different *Sarcocystis* spp. utilize different definitive hosts, such as canids (*S. cruzi*), felines (*S. hirsuta* and *S. bovifelis*), and humans (*S. hominis, S. heydorni, S. sigmoideus*, and *S. suihominis*).

Infection in the intermediate host (herbivore or omnivore) is established when a susceptible animal ingests water or pasture contaminated with sporulated oocysts or sporocysts. In the intestinal lumen, the action of bile salts and proteolytic enzymes, such as trypsin, ruptures the oocyst wall, releasing four motile sporozoites. These invade the endothelial cells of the arteries in the mesenteric lymph nodes, where the first phase of asexual reproduction (schizogony) occurs. This process culminates in the formation of motile merozoites from the primitive schizont, which are subsequently released into the bloodstream between 7 and 14 days post-infection. The merozoites can remain in circulation for 26 to 46 hours, invading the endothelium of arterioles, capillaries, and venules, distributing themselves systemically. Schizogony may also occur in macrophages and parenchymal cells of various organs. The final generation of the schizogonic cycle typically occurs within a myocyte (skeletal, smooth, or cardiac muscle) and, depending on the *Sarcocystis* spp. species, it may affect cells of the central nervous system (CNS)^14,16^. Figure 2 illustrates the life cycle of *Sarcocystis* spp.

Inside the host cell, the tissue cyst (sarcocyst) develops within a parasitophorous vacuole. In this microenvironment, a metrocyte (mother cell) originating from the last generation of merozoites is formed, accompanied by the simultaneous development of a cyst wall that isolates the parasite from adjacent tissues. On average, cysts in the musculature of the intermediate host become infective in two months, although this period varies according to the species. Sarcocysts presents remarkable longevity, remaining viable for years or throughout the entire lifespan of the parasitized host^16^. Cyst distribution and density are influenced by crucial factors such as the host's immunological status, infection stage, parasite species, and the ingested sporozoite load.

Infection of the definitive host occurs by ingesting tissues containing mature cysts (raw, undercooked, or inadequately processed meat). In the gastrointestinal tract, digestion of the cyst wall releases the bradyzoites which penetrate the lamina propria of the intestine and invade the goblet cells and enterocytes of the epithelium. There, gametogony (sexual reproduction) occurs, in which the bradyzoites differentiate into microgametocytes (male) and macrogametocytes (female). Fertilization results in a zygote, around which a thin wall (<1 μm) develops, originating the oocyst. Sporulation occurs *in situ* within the intestinal epithelium, forming two sporocysts, each containing four sporozoites. Given the fragility of the oocyst wall, sporocysts are frequently released individually in the feces, becoming highly infectious environmental sources^16^.

### Clinical manifestations across different hosts

#### Human sarcocystosis (intestinal and muscular)

In humans, sarcocystosis presents in two distinct clinical forms depending on the host's role in the life cycle of the parasite. When humans act as definitive hosts (after ingesting tissue cysts from undercooked meat), they develop intestinal sarcocystosis which presents a clinical picture frequently subclinical and self-limiting. When present, however, the symptoms caused by *S. hominis* and *S. suihominis* include anorexia, dyspnea, tachycardia, vomiting, nausea, abdominal discomfort, and diarrhea. In *S. hominis* infections, eosinophilia is a frequent laboratory finding^11^. Prepatent period, the time elapsed from infection to the shedding of parasitic forms in feces, ranges from 14 to 18 days for *S. hominis* and 11 to 13 days for *S. suihominis* ^10^.

Conversely, when humans act as accidental intermediate hosts (via ingestion of sporocysts from the environment), they develop muscular sarcocystosis. The exact number of *Sarcocystis* spp. that utilize humans as intermediate hosts remains uncertain; however, studies have identified tissue cysts from at least seven different parasite species lodged in human musculature. Muscular sarcocystosis can reach a 3.6% prevalence in Western countries, but the disease gains prominence in Asia, where 21% of the cases are concentrated in Malaysia^17^. Clinical manifestations are more severe, characterized by acute, relapsing febrile myositis, peripheral eosinophilia, and elevated creatine kinase. Notably, *S. nesbitti* has been frequently associated with human outbreaks^15,18^. A documented case in Singapore described a patient presenting with acute muscular sarcocystosis confirmed by open muscle biopsy, Magnetic Resonance Imaging (MRI), and molecular testing (PCR)^18^.

Neurosarcocystosis, a severe central nervous system infection, has been reported in immunocompromised patients. A recent study described an HIV-infected patient in Norway who developed neurosarcocystosis caused by *S. nesbitti*. Diagnosis relied on neurological symptoms, typical lesions on the MRI, and was confirmed via brain tissue metabarcoding and Next-Generation Sequencing (NGS). The authors emphasize that *Sarcocystis* spp. may act as an opportunistic agent in immunocompromised individuals, frequently misdiagnosed due to its clinical similarity to another agent of the Sarcocystidae family, *Toxoplasma gondii* ^19^.

#### Bovine sarcocystosis (acute and chronic phases)

In cattle, which serve as natural intermediate hosts for at least eight different *Sarcocystis* species (*S. cruzi, S. heydorni, S. hominis, S. bovifelis, S. hirsuta, S. bovini, S. rommeli* ^5^ and recently *S. sigmoideus* ^12^), the clinical manifestations depend on the infection phase and the specific agent involved. Among them, *S. cruzi* stands out for presenting the worst prognosis for the herds. Importantly, cattle rarely develop clinical signs during the acute phase. When symptoms do occur, they establish themselves during the second schizogonic cycle in the blood vessels. Symptoms like dyspnea, salivation, opisthotonos, fever, anorexia, alopecia, anemia, nasal discharge, and prostration typically appear three to four weeks after a massive ingestion of oocysts (> 50,000) by the intermediate host. Reproductive issues have also been reported, although rare^14,20,21^.

During the chronic phase, the infection is typically asymptomatic. However, the degeneration of tissue cysts in the musculature can trigger Bovine Eosinophilic Myositis (BEM), a specific inflammatory response to degenerating cysts that results in severe economic losses due to carcass condemnation^10,12^. Some studies suggest that *S. hirsuta* and *S. hominis* may cause this condition, but *S. cruzi* remains the primary agent^22^. The abnormal macroscopic visual appearance makes BEM a recurrent cause of carcass condemnation. In the United States, BEM has been reported as a leading cause of condemnation, accounting for up to a 5% rejection rate^23^. Interestingly, a recent study by Dini *et al*.^24^ described a BEM case in a bovine co-infected by *S. hominis* and *T. gondii*, where molecular analyses confirmed the presence of DNA from both protozoa. The animal's serology was negative for *T. gondii*, suggesting either a recent infection or undetectable antibody levels. This co-infection suggests that *T. gondii* may act as a potentiating factor for the inflammatory response observed in BEM, reinforcing the need for further investigation into the synergistic impact of these parasites on meat sanitary inspection^24^.

#### Equine Protozoal Myeloencephalitis (EPM)

Equines are considered accidental or dead-end intermediate hosts for *S. neurona*, the etiologic agent of Equine Protozoal Myeloencephalitis (EPM). This species is highly pathogenic to several animals such as seals, raccoons, armadillos, and skunks, but especially to equines, due to the formation of schizonts in neural tissue without subsequent cyst development in that site. The main clinical manifestations include dysphagia, abnormal upper airway function, seizures, weight loss, weakness, and gait abnormalities^25^. Expanding the clinical spectrum in companion animals, species like *S. caninum* and *S. svanai* have also been associated with cases of severe myalgia and hepatic injury in dogs across North America^22^.

Another transmission route gaining notoriety involves a 15-kDa toxin originating from *Sarcocystis* spp. cysts. Human food poisoning has been reported in Japan following the ingestion of raw meat from exotic animals such as horses and deer, infected by *S. fayeri* and *S. truncata*, respectively^26,27^. This protein has also been detected in *S. cruzi* cysts in beef, showing homology to the actin-depolymerizing factor of *T. gondii* and *Eimeria tenella* ^27^.

### Epidemiology and zoonotic transmission

#### Global prevalence in herds and human infection

Sarcocystosis presents a worldwide distribution, and *Sarcocystis* spp. is remarkably highly prevalent in livestock, particularly in cattle, with rates ranging from 90% to 100% globally, including in Brazil^28-31^. The parasite possesses a vast homeothermic and poikilothermic profile, having been reported in mammals (74%), birds (14%), reptiles (10%), and fish (0.5%)^32^. In rural environments, various livestock species serve as intermediate hosts, perpetuating specific cycles such as *S. ovicanis* and *S. tenella* in sheep, and *S. miescheriana* and *S. porcifelis* in swine. Regarding definitive hosts, different *Sarcocystis* spp. complete their life cycles in companion animals, especially domestic canids and felids. Canids are particularly noteworthy, as they are considered definitive hosts for *S. miescheriana, S. capracanis, S. arieticanis*, and primarily *S. cruzi* ^10,11^.

The globalized trade in animal products has caused shifts in dietary habits, favouring the consumption of undercooked or raw meat and increasing the risk of infection^1-3^. Studies show that human intestinal sarcocystosis prevalence in Europe is higher compared with other continents. Coproparasitological examinations revealed infection rates of 10.4% in Polish children and 7.3% in Germany^16^. In Asia, the parasite was reported in 42.9% of beef samples in Tibet, with 21.8% of human stool samples positive for *S. hominis* ^33^. In Brazil, a study identified *S. hominis, S. hirsuta*, and *S. cruzi* in 50 raw kibbeh samples from Arab restaurants in Sao Paulo^34^.

This epidemiological ubiquity is further complicated by species that have frequently been misdiagnosed. *S. heydorni*, discovered in 1973 when a researcher in Turkey voluntarily infected himself, was historically erroneously identified as *S. hominis* due to morphological similarities. It was only recognized as a distinct zoonotic species in 2015 based on ultrastructural differences^7^. Data on its global prevalence remain limited (ranging from 0.5% to 0.9% in European cattle), suggesting persistent underreporting^5^. Concurrently, the recently described *S. sigmoideus* (2024), confirmed as zoonotic via high-throughput sequencing of human feces in France^8^, adds another layer of complexity. Its presence was detected in cattle carcasses affected by BEM in Italian slaughterhouses (where BEM prevalence is 0.017%), specifically in 3.9% of extralesional and 4.2% of intralesional samples^8^. Retrospective data suggest its broader prevalence among cattle may range from 1% to 7.7%^22,35,36^. Table 2 summarizes the global distribution and prevalence rates of various *Sarcocystis* species across different regions and hosts.

**Table 2:** Epidemiological prevalence of *Sarcocystis* species in different hosts, meat products, and geographical regions

Region / Country	Host or sample type	*Sarcocystis* species	Prevalence (%)	Article
Global / Brazil	Cattle (Intermediate Host)	*Sarcocystis* spp.	90% – 100%	Hooshyar *et al*.^28^ Ruas *et al*.^29^ Moré *et al*.^30^ Elshahawy *et al*.^31^
Europe (General)	Cattle (Intermediate Host)	*S. heydorni*	0.5% – 0.9%	Dubey *et al*.^5^
Belgium / Italy	Cattle (Intermediate Host)	*S. sigmoideus*	1.0% – 7.7%	Moniot *et al*.^8^ Vangeel *et al*.^22^ Zeng *et al*.^35^ Rubiola *et al*.^36^
Italy	Bovine BEM Carcasses	*S. sigmoideus*	3.9% – 4.2%	Moniot *et al*.^8^
Tibet (Asia)	Beef (Retail Market)	*Sarcocystis* spp.	42.9%	Yu^33^
Tibet (Asia)	Humans (Definitive Host)	*S. hominis*	21.8%	Yu^33^
Poland	Humans (Definitive Host)	*Sarcocystis* spp.	10.4%	Fayer *et al.* ^16^
Germany	Humans (Definitive Host)	*Sarcocystis* spp.	7.3%	Fayer *et al*.^16^
Malaysia	Humans (Intermediate Host)	*S. nesbitti* (Muscular)	21.0%	Poulsen *et al*.^17^
Western Countries	Humans (Intermediate Host)	*Sarcocystis* spp. (Muscular)	3.6%	Poulsen *et al*.^17^

#### Diagnostic challenges and underreporting

Sarcocystosis prevalence in humans remains poorly understood, with most literature reports involving only sporadic disease outbreaks. Several factors contribute to human sarcocystosis going unnoticed by health surveillance programs. Prominent among these challenges are the subclinical nature and incubation period of the parasite, the inefficiency of conventional parasitological methods, and the lack of trained professionals capable of correctly identifying the parasitical structures in different biological samples, especially tissue cysts. Moreover, the absence of a gold-standard diagnostic algorithm for identifying and studying the worldwide prevalence of zoonotic species remains a significant gap. This urgently requires new studies given the emergence of new species that can affect humans, such as *S. heydorni* and *S. sigmoideus* ^7,8^.

#### Meat inspection and regulatory framework in Brazil

From a regulatory perspective, Brazil has a specific Regulation on Industrial and Sanitary Inspection of Animal Products (RIISPOA). This regulation^6^ advocates the use of microscopic and molecular methods for inspecting meat and its by-products to ensure food safety. For cattle infected with *Sarcocystis* spp., RIISPOA Article 168 of Section III mandates the condemnation of carcasses with massive infections, that is, when incisions made in various parts of the animal's musculature present cysts. Otherwise, the carcass may be subjected to conditional utilization^6^. But most *Sarcocystis* spp., including those with zoonotic potential, develop microscopic cysts that frequently go unnoticed during routine sanitary inspection on the slaughter line. Thus, diagnosis of bovine sarcocystosis plays an essential role in preserving the economic value of the meat production chain while safeguarding public health. Rapid and accurate methods like Polymerase Chain Reaction (PCR) enables the identification of early-stage infections, preventing unwarranted condemnations during sanitary inspection. The ability to distinguish between zoonotic species, such as *S. hominis*, and non-zoonotic ones contributes to more efficient herd management, ensuring that healthy carcasses are approved for human consumption^12,37^. Thus, avoiding unnecessary condemnation not only reduces direct financial losses for slaughterhouses but also protects the reputation of Brazilian beef in the global market.

This epidemiological dynamic directly compromises food safety, as failures in sanitary management perpetuate the cycle of the parasite^13^. From the perspective of Public Health and Tropical Medicine, the consequences extend far beyond animal health. Although bovine infection results in carcass condemnation, in accordance with Article 168, and additional costs for producers, the critical risk lies in the inability to detect microscopic cysts during routine visual inspection^6^. This technical limitation allows silently infected meat to enter the consumer chain, perpetuating the risk of human exposure to emerging pathogens and underscoring the urgent need for integrating molecular methods in sanitary surveillance^17^.

#### Diagnosis approaches

To assist in the treatment of human intestinal sarcocystosis caused by *S. hominis, S. heydorni, S. sigmoideus*, and *S. suihominis*, it is advantageous to associate the gastrointestinal clinical manifestations that these species may trigger in humans with the patient's potential dietary habits. However, definitive diagnosis of the disease relies on laboratory tests, particularly the employment of parasitological techniques to identify oocysts and sporocysts in feces. Commonly utilized methods include centrifugal flotation in Sheather's solution, the Faust method, direct fecal smears, and the modified Kato-Katz technique^9,10,14^. Notably, the parasite may go undetected by Ziehl-Neelsen staining, widely used for detecting enteric parasites, because oocysts and sporocysts do not stain consistently with acid-fast staining procedures^17^.

In this scenario, molecular methods emerge as irreplaceable tools. Techniques such as PCR allow for higher sensitivity and specificity, and is fundamental for identifying and differentiating morphologically similar species, such as *Sarcocystis hominis*-like species, or newly described ones like *S. heydorni* and *S. sigmoideus* ^8,12,36^. Such approaches overcome the limitations of diagnoses based exclusively on ultrastructural morphology, which are not always available or detailed for all species, providing the taxonomic precision required by modern epidemiological surveillance. For example, an Italian research group discovered the first partial sequence of the *COX1* gene of *Sarcocystis* spp. present in the feces of human patients with gastrointestinal symptoms, successfully identifying the zoonotic species *S. hominis*. Despite no *Sarcocystis* spp. sporocysts found in the patients’ feces, this study provides promising perspectives for the molecular diagnosis of human intestinal sarcocystosis. Concurrently, the same group developed a multiplex PCR capable of detecting and differentiating various *Sarcocystis* spp. that infect beef, showing the versatility of these tools for sanitary surveillance^36^. Beyond conventional PCR, the development of real-time PCR (qPCR) and next-generation sequencing (NGS) represents the next leap in detecting *Sarcocystis* spp. in beef samples. These technologies allow not only the quantification of the parasite but also the simultaneous detection of multiple species, offering a more comprehensive view of the infection and facilitating continuous large-scale monitoring^8,12,17,36^.

**Figure 3:**
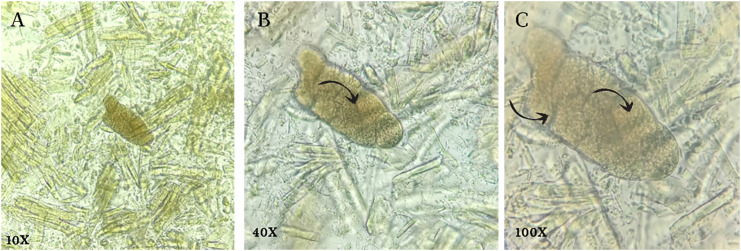
Direct parasitological examination of bovine cardiac muscle tissue containing *Sarcocystis* spp. tissue cysts by conventional light microscopy: (A) Visualization of the intact tissue cyst at 10x magnification; (B) Detailed view at 40x magnification, with the arrow indicating the internal septa; (C) Higher magnification (100x), with the arrow highlighting the septa dividing the cyst into compartments.

**Figure 4:**
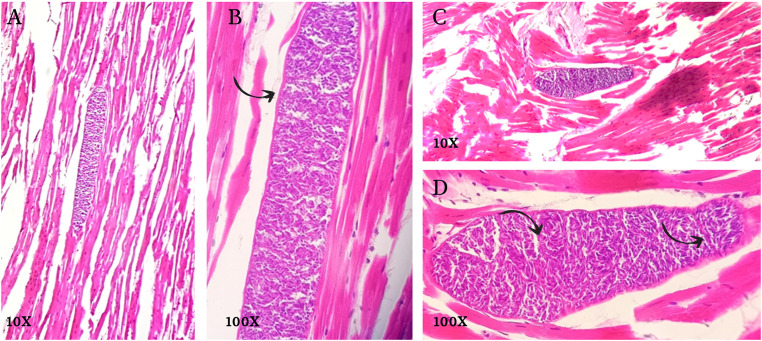
Histological examination of bovine cardiac muscle tissue stained with hematoxylin and eosin (H&E) containing *Sarcocystis* spp. tissue cysts by conventional light microscopy: (A) Overview of the tissue cyst within the muscle fibre at 10x magnification; (B) Detailed view at 100x magnification, where the arrow indicates the cyst wall; (C) Visualization of another tissue cyst at 10x magnification; (D) Detailed view at 100x magnification, with the arrow showing the numerous bradyzoites packed within the tissue cyst.

Muscular sarcocystosis diagnosis is limited, as it depends on identifying tissue cysts in muscle tissue samples obtained by biopsy. Moreover, the probability of obtaining positive samples is low since the diagnosis relies on the biopsy being performed exactly at the site where the tissue cysts are located, which does not occur homogeneously^17^. Direct parasitological approaches using optical or inverted microscopy serve as excellent screening tools for observing *Sarcocystis* spp. tissue cysts due to their characteristic fusiform shape containing internal septa that resemble honeycombs, segregating internal compartments (Figure 3). However, identification via this method is limited to the genus level^38^. In turn, histopathological approaches are useful for differentiating the cyst wall thickness of various *Sarcocystis* spp., but because they are labour-intensive techniques, they prove impractical for large-scale routine application^9^ (Figure 4).

In biopsy material, in addition to *T. gondii*, performing a differential diagnosis for *Trypanosoma cruzi* using Periodic Acid-Schiff (PAS) stain is recommended, particularly in endemic regions such as South America, since *Sarcocystis* spp. and *T. gondii* bradyzoites test positive with this stain, whereas *T. cruzi* does not. Although unfeasible for diagnosing the intestinal form of the disease, serological methods have been proposed for detecting the muscular form. However, most of these tests are currently used for research purposes with limited application in laboratory routines. Prominent among these are the Indirect Enzyme-Linked Immunosorbent Assay (ELISA), Indirect Immunofluorescence Assay (IFA)^39^, and Western Blotting as a confirmatory method^40^.

Moreover, implementing effective diagnostic procedures strengthens epidemiological monitoring, providing crucial data to identify areas of high prevalence of the disease. This information guides strategic investments in sanitary control measures, such as improving water quality and restricting the access of definitive hosts to pastures. By reducing the costs associated with outbreaks and generalized infections, diagnostics directly contribute to the economic sustainability of the sector. This preventive approach, coupled with modern technologies and adequate training, positions molecular diagnosis as an indispensable tool for the competitiveness and efficiency of the meat production chain, ultimately protecting the end consumer^14,41,42^.

### Therapeutic interventions and prophylaxis

#### Pharmacological approaches

Therapeutic approaches for sarcocystosis, both in humans and animals, presents considerable challenges and, in many cases, is either limited or of questionable efficacy. This is largely due to the frequently subclinical nature of the infection, the difficulty in achieving early diagnosis, and the biological complexity of the parasite^10^. Most evaluated antiparasitic drugs target the actively replicating schizonts and are largely ineffective against mature tissue cysts (bradyzoites), which constitute the infective form in meat. Moreover, the availability of specific, licensed medications for treating sarcocystosis in cattle is scarce, underscoring the complexity of directly controlling the disease in already infected herds. The pharmacological arsenal varies significantly depending on the host and the clinical manifestation (such as intestinal vs. muscular forms, or specific conditions like Equine Protozoal Myeloencephalitis)^10,11,31^. Supplementary Table S2 summarizes the main therapeutic options and their respective clinical indications, along with the supporting literature.

#### Prophylaxis and meat safety

Given the therapeutic limitations, prophylactic measures and control of the transmission chain emerge as the most effective and sustainable strategies to mitigate the impact of the disease on Public Health and the agricultural sector. Primary means of preventing infection consists of practicing good hygiene, especially regarding food sanitation and water quality. Notably, conventional water treatment with chlorine does not inactivate the environmental forms (oocysts and sporocysts) of *Sarcocystis* spp., which could explain recurrent outbreaks of human sarcocystosis in Asia^43,44^.

Preventing intestinal sarcocystosis is primarily achieved by cooking meat. Studies have shown that *S. suihominis* cysts in pork can be inactivated at various temperatures and cooking times, for example at 60 °C for 20 min, 70 °C for 15 min, and 100 °C for 5 min.^45^ Moreover, food inspection in slaughterhouses is of paramount importance. A recent study^46^ evaluated the viability of *Sarcocystis* spp. tissue cysts in bovine hearts from Rio Grande do Sul State, Brazil, after freezing treatment. Results revealed that tissue cysts and bradyzoites became non-viable at −35 °C for ≥ 3 h and at −20 °C for ≥ 8 h. This indicates that such thermal treatment configures a viable alternative to the absolute condemnation of bovine carcasses contaminated with *Sarcocystis* spp.

As for transmission of the muscular disease, the main prevention method consists of avoiding the ingestion of water and food contaminated by feces of definitive hosts. Thus, sarcocystosis prevention goes beyond the individual sphere, requiring a collaborative effort between farmers, veterinary health professionals, the food industry, and sanitary authorities. Continuous education on good hygiene practices, control of definitive hosts (such as dogs) on rural properties, and the integration of precise molecular diagnostic methods in sanitary surveillance programs are essential pillars to ensure food safety and the competitiveness of Brazilian beef in the global market^10,11^.

## CONCLUSION

Sarcocystosis is a globally distributed zoonosis that requires further investigation, particularly studies that bring a deeper understanding of the parasite's life cycle, involved hosts, and the environmental factors facilitating its dissemination to other ecological niches. Concomitantly, technology advancement and the emergence of bioinformatics tools has significantly improved the molecular diagnosis of *Sarcocystis* spp., allowing for a better comprehension of interactions between these coccidia and the vast array of hosts they utilize across different biotopes.

From an economic perspective, the livestock and agricultural sectors are essential for Brazil. Moving forward, an integrated One Health approach must be adopted to control this parasitic disease, seeking not only to identify and address its underlying causes but also to promote the health and well-being of all species involved. Reducing sarcocystosis prevalence can increase the market value of cattle and strengthen the competitiveness of Brazilian beef in the global market. Progress in combating sarcocystosis depends on scientific breakthroughs, adequate research funding, robust collaboration across different sectors, and public awareness.

Moreover, the continuous emergence of new *Sarcocystis* spp. with zoonotic potential, such as *S. sigmoideus*, serves as a vivid reminder of the complex dynamics between parasites, hosts, and the environment. This scenario reiterates the urgent need for investments in technological development and human resource training. Only through a holistic approach, one that bridges molecular aspects, ultrastructural studies, and epidemiology with public health and veterinary policies, will be able to advance the mitigation of sarcocystosis, ensure global food safety, and add value to the beef production chain.

## Data Availability

The complete anonymized dataset supporting the findings of this study is available from https://doi.org/10.48331/SCIELODATA.RMSLBR
