# Supplementary material for: Zoonotic potential of bovine Sarcocystis species: a challenge for public health and meat inspection
Source: Rev Inst Med Trop Sao Paulo. 2026 Jul 24;68:e49. doi: 10.1590/S1678-9946202668049 (PMC13405646; doi:10.1590/S1678-9946202668049)
Supplement: Supplementary Material [file 1678-9946-rimtsp-68-S1678-9946202668049-suppl01.pdf]

REVISTA DO INSTITUTO DE MEDICINA TROPICAL DE SÃO PAULO

JOURNAL OF THE SÃO PAULO INSTITUTE OF TROPICAL MEDICINE

SUPPLEMENTARY MATERIAL

https://doi.org/10.48331/SCIELODATA.RMSLBR

Zoonotic potential of bovine *Sarcocystis* species: a challenge for public health and meat inspection

Cayuan Tadeu Brandão Pinto [1], **Luciana Regina Meireles** [1]

Supplementary Table S1 - Chronological timeline of the identification and characterization of *Sarcocystis* spp

Year	Findings
1843	Tissue cysts found in the musculature of house mice[1]
1882	Genus recognized as *Sarcocystis*[2]
1972	Sexual phase cultured *in vitro*[3]
1972	Obligate two-host life cycle recognized[4,5]
1973	Vascular phase recognized and pathogenicity demonstrated[6]
1975	Multiple *Sarcocystis* spp. recognized within a single host[7]
1975	Chemotherapy demonstrated[8]
1976	Abortion due to sarcocystosis recognized[9]
1981	Protective immunity demonstrated[10]
1986	Vascular phase cultured *in vitro*[11]
1989	*Sarcocystis* spp. classified based on cyst wall morphology[12]
1991	Identification of multiple host species for *Sarcocystis* spp., including *S. neurona* as the etiologic agent of Equine Protozoal Myeloencephalitis (EPM)[13]
2015	*Sarcocystis neurona* genome annotated for the first time[14]
2015	Formal description of *Sarcocystis heydorni* as a new species with a bovine (*Bos taurus*) and human (*Homo sapiens*) life cycle[15]
2024	Formal description of *Sarcocystis sigmoideus* as a new species (associated with bovine eosinophilic myositis)[16]
2025	Confirmation of the zoonotic nature of *Sarcocystis sigmoideus* in humans[17]

[1]Universidade de São Paulo, Faculdade de Medicina, Instituto de Medicina Tropical de São Paulo, Laboratório de Protozoologia (LIM-49), São Paulo, São Paulo, Brazil

Correspondence to: Cayuan Tadeu Brandão Pinto
Universidade de São Paulo, Faculdade de Medicina, Instituto de Medicina Tropical de São Paulo, Laboratório de Protozoologia (LIM-49), Av. Dr. Éneas de Carvalho Aguiar, 470, Cerqueira César, CEP 05403-000, São Paulo, SP, Brazil
E-mail: cayuan.tadeu@fm.usp.br

Received: 3 March 2026
Accepted: 27 May 2026

Editor: Thelma Suely Okay

This is an open-access article distributed under the terms of the Creative Commons Attribution License.

Supplementary Table S2 - Therapeutic arsenal and pharmacological approaches for the clinical management of sarcocystosis across different hosts

Host species	Clinical condition	Pharmacological agent(s)	Mechanism of action / Clinical indication	Article
**Humans**	Intestinal sarcocystosis	Cotrimoxazole / Furazolidone	Proposed for specific cases, though often self-limiting; requires symptomatic treatment.	Fayer[10] Dubey *et al*.[14] Spickler[43]
	Muscular sarcocystosis	Albendazole / Metronidazole / Cotrimoxazole + Corticosteroids	Antiparasitic action combined with corticosteroids to manage severe inflammatory responses (myositis/ vasculitis).	Fayer[10] Rosenthal[11] Dubey *et al*.[14]
**Equines**	EPM (*S. neurona*)	Ponazuril / Diclazuril	FDA-approved triazine-based drugs. They target the apicoplast of *S. neurona* schizonts in the CNS.	Dubey *et al*.[14] Reed *et al*.[25] Spickler[43]
	EPM (*S. neurona*)	Sulfadiazine + Pyrimethamine	Synergistic combination targeting the folic acid synthesis pathway of the parasite.	Dubey *et al*.[14] Reed *et al*.[25] Spickler[43]
**Bovines**	Acute sarcocystosis	Amprolium / Salinomycin / Halofuginone	Primarily used prophylactically or to reduce severity during outbreaks. Clinical treatment is rare.	Fayer[10] Rosenthal[11] Dubey *et al*.[14]

References cited in this table correspond to the numbering in the reference list of the main article.

REFERENCES

1. Miescher F. Über eigenthümliche Schläuche in den Muskeln einer Hausmaus. Ber Verh Naturforsch Ges Basel. 1843;5:198-202.
2. Lankester ER. On Drepanidium ranarum, the cell-parasite of the frog's blood and spleen (Gaule's Würmschen). Q J Microsc Sci. 1882;22:53-65.
3. Fayer R. Gametogony of Sarcocystis sp. in cell culture. Science. 1972:175:65-7.
4. Rommel M, Heydorn AO. Beiträge zum Lebenszyklus der Sarkosporidien. III. Isospora hominis (Railliet und Lucet, 1891) Wenyon, 1923, eine Dauerform der Sarkosporidien des Rindes und des Schweins. Berl Munch Tierarztl Wochenschr. 1972;85:143-5.
5. Rommel M, Heydorn AO, Gruber F. Beiträge zum Lebenszyklus der Sarkosporidien. I. Die Sporozyste von S. tenella in den Fäzes der Katze. Berl Munch Tierarztl Wochenschr. 1972;85:101-5.
6. Fayer R, Johnson AJ. Development of Sarcocystis fusiformis in calves infected with sporocysts from dogs. J Parasitol. 1973;59:1135-7.
7. Heydorn AO, Gestrich R, Mehlhorn H, Rommel M. Proposal for a new nomenclature of the Sarcosporidia. Z Parasitenkd. 1975;48:73-82.
8. Fayer R, Johnson AJ. Effect of amprolium on acute sarcocystis in experimentally infected calves. J Parasitol. 1975;61:932-6.
9. Fayer R, Johnson AJ, Lunde M. Abortion and other signs of disease in cows experimentally infected with Sarcocystis fusiformis from dogs. J Infect Dis. 1976;134:624-8.
10. Dubey JP. Development of immunity to sarcocystosis in dairy goats. Am J Vet Res. 1981;42:800-4.
11. Speer CA, Dubey JP. Vascular phase of Sarcocystis cruzi cultured in vitro. Can J Zool. 1986;64:209-11.
12. Dubey JP, Speer CA, Fayer R. Sarcocystosis of animals and man. Boca Raton: CRC Press; 1989. p.15.
13. Dubey JP, Davis SW, Speer CA, Bowman DD, De Lahunta A, Granstrom DE, et al. Sarcocystis neurona n. sp. (Protozoa: Apicomplexa), the etiologic agent of equine protozoal myeloencephalitis. J Parasitol. 1991;77:212-8.
14. Blazejewski T, Nursimulu N, Pszenny V, Dangoudoubiyam S, Namasivayam S, Chiasson MA, et al. Systems-based analysis of the Sarcocystis neurona genome identifies pathways that contribute to a heteroxenous life cycle. mBio. 2015;6:e02445-14.
15. Dubey JP, van Wilpe E, Calero-Bernal R, Verma SK, Fayer R. Sarcocystis heydorni, n. sp. (Apicomplexa: Sarcocystidae) with cattle (Bos taurus) and human (Homo sapiens) cycle. Parasitol Res. 2015;114:4143-7.
16. Rubiola S, Moré G, Civera T, Hemphill A, Frey CF, Basso W, et al. Detection of Sarcocystis hominis, Sarcocystis bovifelis, Sarcocystis cruzi, Sarcocystis hirsuta and Sarcocystis sigmoideus sp. nov. in carcasses affected by bovine eosinophilic myositis. Food Waterborne Parasitol. 2024;34:e00220.
17. Moniot M, Combes P, Costa D, Argy N, Durieux MF, Nicol T, et al. Simultaneous Detection of Sarcocystis hominis, S. heydorni, and S. sigmoideus in Human Intestinal Sarcocystosis, France, 2021-2024. Emerg Infect Dis. 2025;31:559-63.
